# When Do Newborns Die? Timing and Cause-Specific Neonatal Death in Neonatal Intensive Care Unit at Referral Hospital in Gedeo Zone: A Prospective Cohort Study

**DOI:** 10.1155/2020/8707652

**Published:** 2020-02-15

**Authors:** Akine Eshete, Sileshi Abiy

**Affiliations:** ^1^College of Health Sciences and Medicine, Department of Public Health, Debre Berhan University, Ethiopia; ^2^College of Health Sciences and Medicine, Department of Anesthesiology, Dilla University, Ethiopia

## Abstract

**Background:**

Maternal, newborn, and child health have a high stake in the global health agenda, however, neonates' risk of dying is unacceptable in the world. Ethiopia is one of the countries with high burden of neonatal morbidity and mortality. Yet, timing and cause-specific neonatal death are under-investigated. The present study aimed to determine the timing and cause-specific neonatal death.

**Methods:**

We conducted a prospective cohort study at Dilla University Referral Hospital Neonatal Care Unit from November 2016 to January 2018. All admitted neonates to the neonatal care unit were followed from cohort entry up to the occurrence of an event (death) or end of follow-up. We generated descriptive statistics to determine the timing of neonatal death and the cause of deaths.

**Results:**

Overall, 11.6% of neonates died during the follow-up. We found that 34.0% and 64.3% of deaths occurred during the first and second weeks of neonatal life, respectively. Neonatal sepsis and low birth weight were the main causes of death and hospital admission. Jaundices and low birth weight were the most common causes of death during the early neonatal period, whereas birth asphyxia, low birth weight, and sepsis were during late neonatal life. However, for other causes of death, the slight difference was seen between the death patterns in early and late neonatal periods.

**Conclusions:**

The timing and cause-specific neonatal deaths were varying among different time of the neonatal periods that needs to design context-based policy and interventions.

## 1. Background

Newborn health has improved significantly in the era of the Millennium Development Goals (MDGs), even though the target of reducing neonatal mortality by two-thirds was not achieved [1], with an estimated 2.6 million newborns dying within 28 days of life and 7000 newborns dying each day around the world in 2016 [2]. Moreover, neonatal mortality reduction has slower progress around the world [3–5], particularly in sub-Saharan Africa, where the reduction is too slow and the sharing of death constitutes for 35% from all under-five children deaths in the world [3]. During the neonatal periods, the risk of neonatal deaths were higher [6–8].

Ethiopia had one of the world's highest neonatal mortality rate (NMR) (29 per 1000 live births) in 2016 [9, 10]. Their NMR was higher than that of the African continent (27 deaths per 1000 live births) and nine times higher than that of the developed countries (3 deaths per 1000 live births); however, it was similar to the sub-Saharan African countries (29 deaths per 1000 live births) [4, 11].

Maternal, newborn, and child health have a high stake in the global health agenda including Ethiopia; however, millions of newborn babies are dying as a result of easily preventable causes [12–14]. Infectious diseases (sepsis, meningitis, and pneumonia), preterm birth complications, and intrapartum complications (including asphyxia) are the leading causes of death in the world [12, 15].

Evidence indicates that 77.5% of neonatal deaths are caused by preterm complications [16] and from 2.7 million newborn deaths about 60-80% of deaths are caused by prematurity [17]. In other evidence from Africa, Asia [18], India [19, 20], and Ethiopia [21], prematurity, neonatal infections, and birth asphyxia were the main causes of deaths. The causes of neonatal death vary among different socioeconomic strata and geographic regions in Ethiopia [22–25].

As a result, countries with high neonatal death are advised to scale up child survival programs in line with their cause-specific deaths [1, 16, 26]. Yet, countries, particularly in sub-Saharan Africa including Ethiopia, have limitations for timing and cause-specific death analysis [16]. Understanding the cause-specific neonatal death gives an important public health insight [4] and also contributes to ending preventable newborn deaths [27, 28], such as reducing preterm-related deaths by 58%, intrapartum-related deaths by 79%, and infection-related deaths by 84% [29]. Additionally, providing quality care at the time of pregnancy and labor and immediate postpartum care have been shown to avert 92% of newborn deaths [15, 30].

Therefore, it is advisable to produce data on the timing of neonatal death and cause-specific death-related information to guide policy and decision-makers. To the best of our knowledge, there is a paucity of reliable information on timing and cause-specific neonatal death in the study area. Therefore, this current study aimed to determine timing of death and cause-specific neonatal death in the first 28 days of life.

## 2. Methods

### 2.1. Study Design, Setting, and Populations

A prospective cohort study was conducted among neonates admitted to the Dilla University Referral Hospital Neonatal Intensive Care Unit (NICU) from November 2016 to January 2018. Dilla University Referral Hospital serves as a referral hospital for all districts in the Gedo zone and Abay district of the Oromia region and the Dara district in the Sidama zone. In the Gedo zone, only the Dilla University Referral Hospital Neonatal Intensive Care Unit is established. All neonates admitted to the neonatal care unit from Dilla Zuria, Wonago, Bule, Gedeb, Kochere, Yirgachefee, and Dilla town, Abay and Dara districts were followed from cohort entry up to the occurrence of an event (death) or end of follow-up.

### 2.2. Recruitment and Interviewing of Study Participants

A structured and interview-administered questionnaire adopted from previous research [22] and WHO questionnaires [31] were used to prospectively collect the data. The mothers of index neonates were interviewed in addition to the data collected from the clinical chart. Ten trained data collectors interviewed mothers of index children and gathered clinical information by reviewing their clinical charts. They followed the neonates from admission to death or discharge.

### 2.3. Data Management and Analysis

In this study, cause-specific death was defined as the death of a neonate during the first 28 days of life attributed to a specific cause [22]. Early neonatal mortality was defined as the probability of death before 7 completed days of life, and late neonatal mortality was defined as the probability of dying between 7 completed days and before 28 completed days. The outcome variables were the timing of death and cause-specific neonatal deaths in the first 28 days of life. A final assessment of admission diagnosis and causes of death were set by physicians after conducting the necessary clinical and laboratory investigation based on national guidelines. Causes of deaths were extracted from the medical records during follow-up.

We checked the data integrity using EpI INFO version 7 and analyzed using statistical package for social sciences (SPSS) version 21. We generated descriptive statistics using the frequency distribution for categorical variables and using mean for continuous variables. We generated descriptive statistics to determine the timing of neonatal death and the cause of deaths.

## 3. Results

### 3.1. Characteristics of Mothers and Neonates

Overall, 987 neonates were admitted to the NICU of Dilla University Referral Hospital. We included 913 neonates into the analysis and excluded those newborns with age greater than 28 days (*n* = 74) at the time of admission. The majority of mothers (*n* = 341, 37.3%) were in the age group of 24-29 years in the last birth with a mean (±SD) age of 26.6 (±4.7) years. Three-fourths of the mothers (75%) completed primary and high school education, and about 58.6% of them were housewives.

Even though most of the mothers (*n* = 776, 85%) reported to have at least one antenatal care (ANC) visit during their last pregnancy, more than half of them (*n* = 518, 56.7%) had less than four ANC visits (focused ANC). About 54.3% (*n* = 496) of the women gave their last birth at government hospitals, while 327 (35.8%) of the women's last delivery were at primary health care centers.

There was a male predominance among the admitted neonates in the NICU (*n* = 510, 55.9%). Most of the admissions (86.3%) occurred during the first week of life. Above two-thirds of neonates were term babies (67.5%), while preterm babies were 32.5%. The mean weights of neonates at admission and discharge was 2737.7 (SD = ±839.8) and 2878.8 (SD = ±1031) grams, respectively (Table 1).

### 3.2. Magnitude and Timing of Death among Admitted Neonates

**Figure 1:**
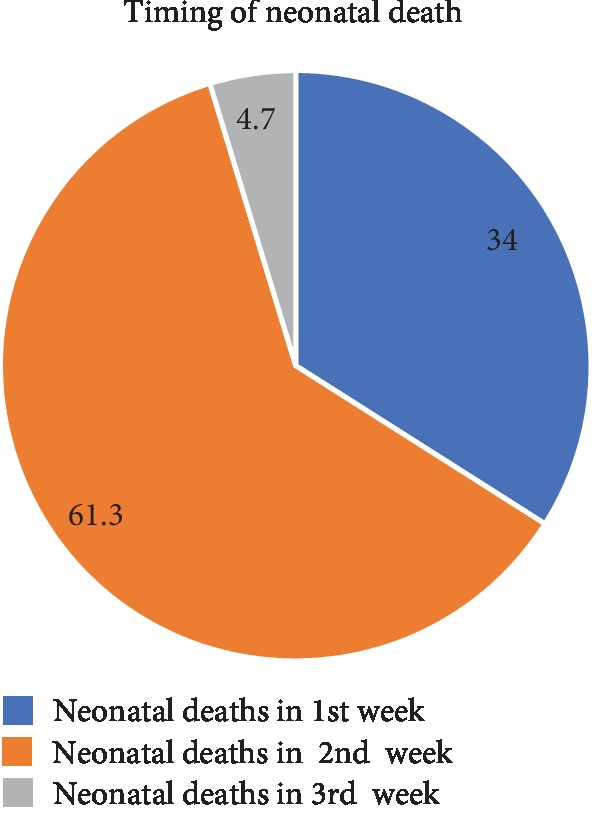
Magnitude and timing of neonatal death in Gedeo zone, Southern Ethiopia, from November 2016 to January 2018.

Overall, 106 neonates died during follow-up, which makes a neonatal mortality of 11.6% (*n* = 106/913), with 95% CL (9.6-13.7). The majority of neonatal deaths occurred during the first 34.5% with (95%CI: 25.5-43.4) and second 61.3% with (95%CI: 51.9-69.8) weeks of neonatal life. Late neonatal death 66.0% with (95%CI: 56.6-74.5) were higher compared to early neonatal deaths 34.5% with (95%CI: 25.5-43.4) (Figure 1).

### 3.3. Cause-Specific Neonatal Death during Follow-Up

**Figure 2:**
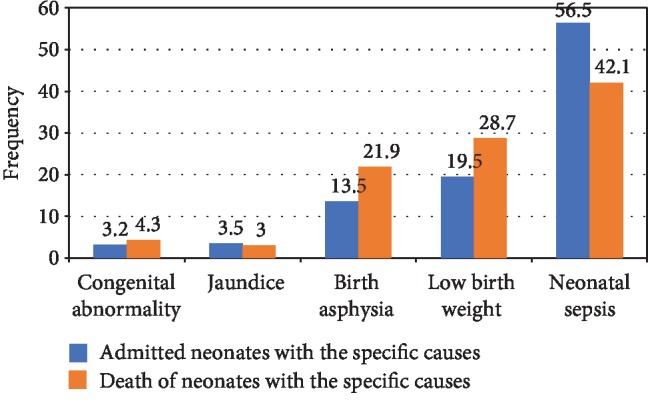
Clinical diagnosis and contribution to deaths in Gedeo zone, Southern Ethiopia, from November 2016 to January 2018. A neonate having more than one diagnosis is counted more than once.

Sepsis (*n* = 98, 42.1%), low birth weight (LBW) (*n* = 67, 28.8%), and birth asphyxia (*n* = 51, 21.9%) were the leading cause of deaths. Birth asphyxia was not the major cause of admission, but it accounted for a higher proportion (21.9%) of neonatal death (Figure 2).

### 3.4. Timing and Cause-Specific Neonatal Death during Follow-Up

Low birth weight (LBW) (*n* = 26, 38.8%) and neonatal sepsis (*n* = 35, 35.7%) were the most common causes of death for the first week of life, whereas sepsis (*n* = 62, 63.3%), birth asphyxia (*n* = 33, 64.7%), and LBW (*n* = 38, 56.7%) were the main causes of deaths for the second week of life. The proportion of deaths from jaundices was relatively higher in the first and second weeks of neonatal life (Table 2).

In the early period, jaundices (*n* = 3, 42.9%) and LBW (*n* = 26, 38.8%) accounted for higher deaths, while in the late neonatal period, birth asphyxia (*n* = 37, 72.5%) and prematurity (*n* = 41, 65.1%) accounted for higher death. Congenital abnormality was not the major cause of death in the early period, but it accounted for a higher proportion of neonatal deaths in the late neonatal period (Table 2).

## 4. Discussion

In the present study, 11.6% of neonates died during follow-up. We found that most of the deaths occurred during the first (34.0%) and second (61.3%) weeks of neonatal life, which were in line with the previous systematic review studies [7, 8] and the studies done in India [32] and Tanzania [33] and also the studies done in Ethiopia [10, 22–24].

Neonatal sepsis and LBW were the leading causes of neonatal deaths in the study. Similar trends were observed in many other countries including Ethiopia [1, 10, 18, 20–24]. This higher mortality related to sepsis and LBW may be associated with poor obstetric care practice during labour and at the time of birth and poor quality special care for sick and small newborns. This could be an alarm to health professionals to improve the quality care at the time of birth and special care for sick and small newborns. This requires responsive health systems that are equipped with lifesaving commodities and well-trained staff such as pediatricians, neonatologists, and neonatal nurses for small and ill newborn babies.

In this current study, birth asphyxia is not the major cause of admission, but it attributed to high number of death, which was in line with previous study findings in Ethiopia and abroad [1, 22, 23, 25, 28]. These findings showed that mortality associated with birth asphyxia may be related to inadequate care for women during labour and basic neonatal resuscitation. Therefore, intrapartum monitoring of high-risk pregnancy and care at the time of birth including adequate basic neonatal resuscitation are the highest priority interventions to reduce neonatal death associated with birth asphyxia.

In the study, low birth weight (38.8%) and jaundices (42.9) were the leading causes of death during the early neonatal period, whereas birth asphyxia (72.5%), neonatal sepsis (64.3), and LBW (61.2%) were during late neonatal life in agreement with previous studies [6–8, 32, 33] and the studies done in Ethiopia [22–24]. Therefore, this finding indicates that improving the survival of LBW neonates is a particular challenge in the study area, similar to other countries [4, 18, 25, 34]. Hence, emphasis must be placed on the best possible basic care of these neonates. However, our hospital is working in a climate of inadequate provision of equipment and well-trained health care workers.

In the early and late neonatal period, poor maternal health condition monitoring could contribute to high deaths. The study finding showed that early and late neonatal deaths were higher where mothers attended fewer (<4) ANC visits, did not have ANC visits, and mothers who were not visited by health extension workers. Several previous studies [35–37] have also reported that lack of ANC visit and quality care results in inadequate monitoring of pregnancy and maternal and neonatal complications during and after delivery, and these are associated with increased risk of neonatal death. Therefore, this finding suggests that special consideration needs to be given for maternal obstetric care during pregnancy.

In the current study, in both the early and late neonatal periods, neonatal death was higher in rural areas compared to an urban area. The reason may be the mothers who lived in the urban area have awareness of the advantage of maternal health care service utilization to them and their babies during pregnancy, labor, immediate and early neonatal periods.

With the presence of some limitations in the study, the finding was generally consistent with those studies in Ethiopia and abroad. In this study, only admitted neonates at Dilla University Referral Hospital were involved, and this population may not be representative of other hospitals and health centers in Ethiopia. However, this present study determines the timing of death and cause-specific death in the first 28 days of life.

## 5. Conclusion

In the present study, 11.6% of neonates died during follow-up period. Neonatal sepsis, LBW, and birth asphyxia were the predominant causes of death and hospital admission. We found that the majority of deaths occurred during the first (34.0%) and second (61.3%) weeks of neonatal life. Jaundices (42.9), low birth weight (38.8%), and sepsis (35.7%) were the leading causes of death in the early neonatal period, whereas birth asphyxia (72.5%), neonatal sepsis (64.3), and LBW (61.2%) were during the late neonatal period.

However, the observed leading causes of death and admission related to maternal and neonatal characteristics could be improved by providing a continuum of care to women during pregnancy, labor, and immediate and early neonatal periods [35, 38]. Besides, a responsive health care system that is equipped with lifesaving commodities and well-trained staff is the priority intervention in our setup.

## Figures and Tables

**Table 1:** Selected sociodemographic and reproductive characteristics of mothers and neonates in Gedeo zone, Southern Ethiopia, from November 2016 to January 2018.

Variables		Number (%)
Age of mothers at the current pregnancy	Mean (SD)	26.6 (±4.7)
	≤24 years	287 (31.4)
	25-29 years	341 (37.3)
	>30	285 (31.2)

Districts of the respondents	Dilla Zuria	119 (13.0)
	Wonago	108 (11.8)
	Bule	81 (8.9)
	Gedeb	79 (8.7)
	Kochere	84 (9.2)
	Yirgachefee town and district	131 (14.3)
	Dilla town	154 (16.9)
	Abay district, Oromia region	82 (9.0)
	Dara district, Sidama zone	75 (8.2)

Place of residence	Urban	316 (34.6)
	Rural	597 (65.4)

Mother's educational status	Uneducated	168 (18.4)
	Primary school (grades 1-8)	324 (35.5)
	High school (grades 9-12)	362 (39.5)
	Diploma and above	59 (6.5)

Mothers' occupational status	Government or private employee	52 (5.7)
	Self-employed including merchant	326 (35.7)
	Housewife	535 (58.6)

Family size	Mean (SD)	4.01(±2.3)
	Less than three family	495 (54.5)
	4-6 family	272 (29.8)
	Greater than seven family	146 (16.0)

ANC visit in the last pregnancy	<4 ANC visits	518 (56.7)
	>4 ANC visits	258 (28.3)
	No ANC visit	137 (15)

HEW visit during pregnancy	1-2 visits	132 (14.5)
	≥3 visits	31 (3.4)
	Not visited by HEW	750 (82.1)

Place of delivery	Hospital	496 (54.3)
	Health center	327 (35.8)
	Private clinic	30 (3.3)
	Home	60 (6.6)

Delivery assistant	Skilled personnel	848 (92.9)
	Family or relatives and TBA	65 (7.1)

Age of the neonates at admission (days)	Mean (SD)	4.4 (±4.03)
	1-7 days	788 (86.3)
	7-14 days	79 (8.7)
	14-21 days	40 (4.4)
	21-28 days	6 (0.7)

Sex of neonate	Male	510 (55.9)
	Female	403 (44.1)

Weight of the neonates at birth	Mean (SD)	2639.1 (±818.4)
	<2500 grams	344 (37.7)
	≥2500 grams	569 (62.3)

Gestation age at birth	Mean (SD)	37.2 (±2.4)
	Preterm (<37 weeks	297 (32.5)
	Term (>37 weeks)	616 (67.5)

SD: standard deviation; TBA: traditional birth attendant; ANC: antenatal care; HEW: health extension worker.

**Table 2:** Cause-specific numbers of neonatal deaths and proportions in Gedeo zone, Southern Ethiopia, from November 2016 to January 2018.

Diagnosis of causes^∗^	No. of neonates admitted	Number of deaths	Time of death		
			Neonatal deaths in 1st week, *n* (%)	Neonatal deaths in 2nd week, *n* (%)	Neonatal deaths in 3rd week, *n* (%)
Sepsis	835	98	35 (35.7)	62 (63.3)	1 (1.0)
Low birth weight	344	67	26 (38.8)	38 (56.7)	3 (4.5)
Birth asphyxia	199	51	14 (27.5)	33 (64.7)	4 (7.8)
Jaundice	52	7	3 (42.9)	3 (42.9)	1 (14.3)
Congenital abnormality	48	10	1 (10.0)	9 (90.0)	—
Total, % of deaths	913	106	36 (33.9)	65 (61.3)	5 (4.7)
Diagnosis of causes^∗^	No. of neonate admitted	Number of death	Time of death		
			Early neonatal period	Late neonatal period	
Sepsis	835	98	35 (35.7)	63 (64.3)	
Low birth weight	344	67	26 (38.8)	41 (61.2)	
Birth asphyxia	199	51	14 (27.5)	37 (72.5)	
Jaundice	52	7	3 (42.9)	4 (57.1)	
Congenital abnormality	48	10	1 (10.0)	9 (90.0)	
Total, % of deaths	913	36 (33.9)	65 (61.3)	5 (4.7)	

^∗^A neonate having more than one diagnosis is counted more than once; therefore, the proportion of deaths exceeds 100%.

## Data Availability

The authors confirm that all relevant data were included in the manuscript and the raw data set can be obtained by email request at akine.eshete@yahoo.com.

## Abbreviations

ANC: Antenatal care
LBW: Low birth weight
MDGs: Millennium Development Goals
NICU: Neonatal intensive care unit
NMR: Neonatal mortality rate
SPSS: Statistical Package for Social Sciences
SD: Standard deviation
WHO: World Health Organization.
